# Increased Reactive Oxygen Species and Distinct Oxidative Damage in Resveratrol-suppressed Glioblastoma Cells

**DOI:** 10.7150/jca.45489

**Published:** 2021-01-01

**Authors:** Bin Jia, Xu Zheng, Mo-Li Wu, Xiao-Ting Tian, Xue Song, Yan-Na Liu, Pei-Nan Li, Jia Liu

**Affiliations:** 1 Liaoning Laboratory of Cancer Genetics and Epigenetics and Department of Cell Biology, College of Basic Medical Sciences, Dalian Medical University, Dalian 116044, China.; 2 Department of Orthopaedic Surgery, Second Affiliated Hospital, Dalian Medical University, Dalian 116011, China.

**Keywords:** resveratrol, glioblastoma multiforme, reactive oxygen species, oxidative activity, mitochondrial damage

## Abstract

**Background and Aim:** Glioblastoma multiforme (GBM) is a highly aggressive brain malignancy that lacks reliable treatments. Resveratrol possesses anti-cancer effects, but its activity against glioblastoma cells is variable for unknown reasons. One mechanism through which anti-cancer drugs exert their effects is oxidative damage caused by increased reactive oxygen species (ROS) production. Thus, the present study examined the relationship between oxidative stress and sensitivity to resveratrol in glioblastoma cells.

**Methods:** Two GBM cell lines (U251 and LN428) were exposed to 100 μM resveratrol for 48 h, and proliferation and apoptosis were assessed. ROS generation was evaluated using 2′-7′-dichlorodihydrofluorescein diacetate-based flow cytometry and fluorescent microscopy. Immunocytochemical staining and western blotting were conducted at regular intervals to profile the expression patterns of superoxide dismutase-2 (SOD2), catalase, caspase-9, caspase-3, and sulfotransferases (SULTs) in untreated and resveratrol-treated GBM cells.

**Results:** Resveratrol-treated U251 cells, but not resveratrol-treated LN428 cells, exhibited remarkable growth arrest and extensive apoptosis accompanied by elevated intracellular ROS levels and attenuated SOD2 and catalase expression. Mitochondrial impairment and more distinct increases in the expression of activated caspase-9 and caspase-3 were detected in U251 cells following resveratrol treatment. The levels of resveratrol metabolic enzymes (SULT1A1 and SULT1C2) were lower in U251 cells than in LN428 cells.

**Conclusions:** Resveratrol increased ROS generation and induced oxidation-related cellular lesions in U251 cells by activating an ROS-related mitochondrial signal pathway. The levels of SULTs and ROS may indicate the therapeutic outcomes of resveratrol treatment in GBM.

## Introduction

Glioblastoma multiforme (GBM) is the most common brain malignancy in adults 1. Despite recent advances in aggressive surgery combined with adjuvant therapies 2, the prognosis of advanced GBM remains extremely poor because most patients with GBM die within 15 months after diagnosis and fewer than 3%-5% of patients survive for more than 5 years 3. Therefore, more effective, less toxic anti-cancer agents that can penetrate the blood-brain barrier are urgently needed.

Resveratrol (3,5,4′-trihydroxystilbene) has a wide range of health benefits including chemopreventive, anti-inflammatory, anti-oxidant, and anti-cancer activities 4. The chemopreventive and anti-cancer effects of this agent have been demonstrated in several cancer types 5. More importantly, resveratrol can penetrate the blood-brain barrier via simple diffusion, indicating its therapeutic potential in the treatment of brain malignancies 6, 7. Our previous data revealed that human GBM cells responded differently to resveratrol 8. For instance, U251 cells were sensitive to 100 μM resveratrol in terms of growth arrest and apoptotic cell death, whereas LN428 cells did not respond to the same treatment 8. Thus, it is important to investigate the underlying factors that explain the differential resveratrol responsiveness of glioblastoma cells for personalized therapy.

Reactive oxygen species (ROS), a group of highly reactive ions and molecules, are generated in the mitochondria as oxygen metabolism by-products 9 and recognized as powerful signaling molecules involved in the cell crisis caused by anti-cancer drugs 10. In cancer cells, higher ROS levels result in oxidative damage in mitochondria and mitochondrial alterations that trigger the apoptosis cascade by releasing apoptotic signals. Redox regulation plays critical roles in maintaining the survival of malignant cells by reducing ROS levels 11. Therefore, ROS levels and the efficiency of their dynamic regulation may determine the response of cancer cells to chemotherapy 12-14. Resveratrol has been reported to have anti-oxidant activity in normal cells, but less data are available for cancer cells 15. Recently, we found abundant spheroid mitochondria, an indicator of oxidative mitochondrial damage, in resveratrol-treated ovarian cancer cells 16. Our previous studies revealed that the levels of resveratrol-related metabolic enzymes such as sulfotransferases (SULTs) are lower in cancer cells than in their normal counterparts. Moreover, SULT levels are negatively correlated with the sensitivity of glioblastoma cells to resveratrol 17. These phenomena may explain why resveratrol exerts beneficial effects on normal cells but damaging effects on malignant cells 18. It is therefore possible that resveratrol increases rather than reduces oxidative stress in cancer cells, presumably because of the poorly operated intracellular resveratrol metabolic machinery in cancer cells. This study examined this speculation using resveratrol-sensitive U251 and resveratrol-resistant LN428 cells.

## Materials and Methods

### Chemicals and antibodies

Resveratrol, dimethylsulfoxide (DMSO) and methylthiazolyldiphenyl-tetrazolium bromide (MTT) were purchased from Sigma-Aldrich Co (St. Louis, MO, USA). TUNEL (terminal dexynucleotidyl transferase (TdT)-mediated dUTP nick end labeling) kit was purchased from Roche Inc., Germany. 2'-7'-dichlorodihydrofluorescein-diacetate (DCFH-DA) was purchased from Beyotime Institute of Biotechnology (Jiangsu, China). The rabbit anti-human antibodies against SOD2, Catalase, SULT1A1 and SULT1C2 were purchased from Protein Tech (Chicago, IL, USA), and the rabbit anti-human pro-caspase-3, active-caspase-3, pro-caspase-9 and active-caspase-9 antibodies were provided by Abcam (Cambridge, UK).

### Cell lines and cell culture

Human glioblastoma LN428 cell lines were kindly provided by Professor Nicolas de Tribolet, Department of Neurosurgery, Central Hospital University of Laussane, Switzerland and human glioblastoma U251 cell line was provided by the Cell Culture Facility, Chinese Academy of Sciences Cell Bank, Shanghai. The cells were cultured in DMEM (Hyclone Lab, Utah, USA) supplemented with 10% fetal bovine serum (Gibco Life Science, Grand Island, NY, USA), 100 IU/ml penicillin, and 100 μg/ml streptomycin under 5% CO_2_ and 37 °C condition. The cells (5×10^4^/ml) were cultured conventional dishes for protein preparation or seeded to the high throughput coverslip preparation dishes (Jet Biofil Biotech Inc., Guangzhou, China) to prepare sufficient cell-bearing coverslips for different experimental purposes. 100 μM resveratrol was used to treat the cells.

### Cell proliferation and death assays

Hematoxylin-eosin (H/E) morphological staining was performed on the cell-bearing coverslips with different treatments. The effects of resveratrol on cell proliferation were determined at 24 hour and 48 hour time points by 3-[4,5-Dimethylthiazol-2-yl]-2,5-diphenyl-tetrazolium bromide (MTT) assay and shown in OD values. In paralell, TUNEL assay was employed to detect apoptotic cells according to producer's instructions (Promega Corporation, USA).

### Ultrastructural examination

U251 and LN428 cells without and with 48 h 100 μM resveratrol treatment were harvested and fixed in 2.5% glutaraldehyde (30min, 50 mM cacodylate buffer, pH 7.2) and 2% OsO4 (30 mimutes, same buffer). Ultra-thin sections (0.1 μM) were prepared and examined under a Philips CM100 transmission electron microscope (FEI Company, USA). Images were captured by charge-coupled device camera equipped with TCL-EM-Menu version 3 from Tietz Video and Image Processing Systems (Gaunting, GmbH, Friedrichshafen, Germany) as described elsewhere 18.

### Intracellular ROS determination

2'-7'-dichlorodihydrofluorescein-diacetate (DCFH-DA) penetrates cells and becomes hydrolyzed to non-fluorescent dichlorodihydrofluorescein (DCFH). DCFH reacts with ROS to form the highly fluorescent dichlorofluorescein (DCF) which can be measured by a flow cytometry. Briefly, U251 and LN428 cells were treated with 100 μM resveratrol and collected at 0 h, 6 h, 12 h, 24 h and 48 h time points. The cells (2×10^5^) were incubated with 10 μM DCFH-DA (Beyotime Biotech, Jiangsu, China) for 20 minutes at 37 °C in dark room and washed twice with DMEM. ROS levels were analyzed using a flow cytometer (FACSCalibur, BD Biosciences, San Diego, CA, USA). The cell-bearing coverslips collected at the same time were stained *in situ* with DCFH-DA and observed and photographed under a fluorescence microscope (Leica, DMI4000B, Germany).

### Immunocytochemical staining

Immunocytochemical staining (ICC) was performed by the method described elsewhere 16. The rabbit anti-human SOD2, Catalase, rabbit SULT1A1 and SULT1C2 (Proteintech, Chicago, IL, USA) were used in the dilution rates of 1:500, 1:500, 1:200, 1:150, respectively. Color reaction was developed using 3, 3'-diaminobenzidinete-trahydrochloride (DAB). According to the labeling intensity, the staining results were evaluated by two independent researchers and scored as negative (-) if no immunolabeling was observed in target cells, weakly positive (+), moderately positive (++), and strongly positive (+++).

### Western blot analysis

Total cellular proteins were prepared from the cells by the method described previously 17. 30 μg sample proteins were separated with 12% SDS/PAGE, and transferred to a polyvinylidene difluoride membrance (Amersham, Buckinghamshire, UK). The membrance was blocked with 5% skimmed milk in NaCl/Tris-T (10 mM Tris/HCl, pH 8.0, 150 mM NaCl, and 0.5% Tween-20) at 4 °C overnight, incubated for 2 hours with the primary antibody and then with horseradish peroxidase-conjugated anti-rat IgG (Zymed Laboratories, San Francisco, CA, USA) for one hour. Immunolabeling was detected with an enhanced chemiluminescence system (Roche Inc., Mannheim, Germany), and visualized with the UVP Bio-spectrum Imaging System (UVP, Upland, CA, USA). β-actin was used as the internal quantitative control in densitometry analyses.

### Statistical analysis

The experiments were repeated at least for three times and the the normality of the data obtained were analyzed using SPSS software (version 17.0; SPSS, Chicago, IL). The differences in continuous variables were assessed by Student's t-test or one-way ANOVA. Values are presented as the mean ± standard deviation of triplicate experiments. When required, *P*-values are stated in the figure legends.

## Results

### Different resveratrol sensitivities of U251 and LN428 cells

**Figure 1:**
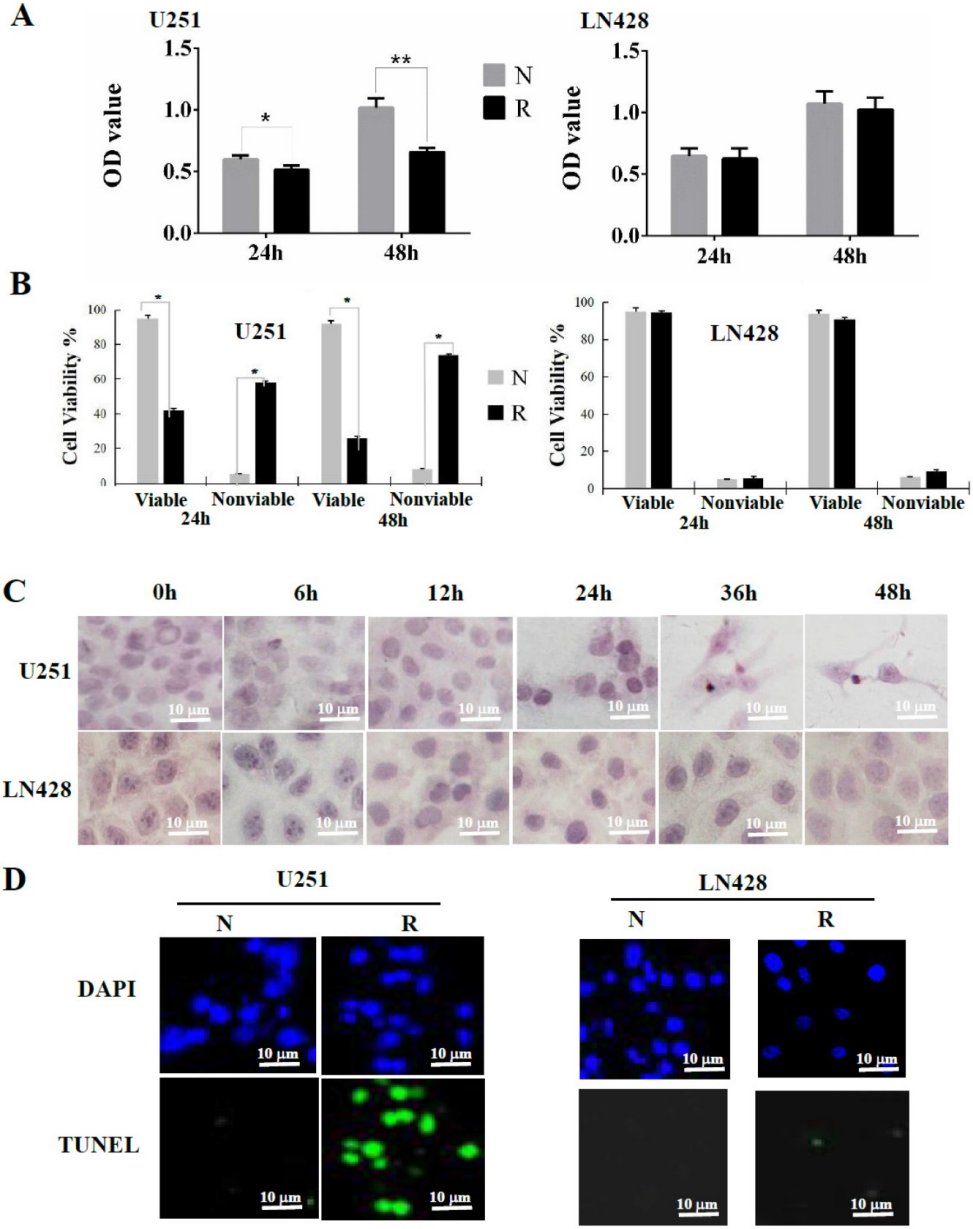
** Evaluation of resveratrol sensitivities of U251 and LN428 cells.** Resveratrol sensitivities of U251 and LN428 cells were evaluated by MTT assay (**A**), hematoxylin and eosin morphological staining (**B**) and fluorescent TUNEL labeling (**C**). N, without resveratrol treatment; R, treated by 100 µM resveratrol. *, *P* <0.05 in comparison with N group; **, *P* <0.01 in comparison with N group.

The MTT cell proliferation assay revealed that the optical density (OD) of 100 μM resveratrol-treated U251 cells was reduced in a time-dependent manner (24 h, *P* < 0.05; 48 h, *P* < 0.01) in comparison with that of the control cells cultured in medium containing 0.2% DMSO (**Figure 1A**). The OD of LN428 cells treated with 100 μM resveratrol for 48 h was reduced by 4.3% compared with that in control cells (*P* > 0.05).

### Extensive apoptosis of resveratrol-treated U251 cells

A cell viability assay revealed a time-dependent increase of the nonviable fraction of resveratrol-treated U251 cells, but not in LN428 cells (**Figure 1B**). Cytopathological staining using hematoxylin and eosin revealed a distinct apoptotic phenotype in resveratrol-treated U251 cells but not in drug-treated LN428 cells, including cellular shrinkage, chromatin condensation, and the appearance of apoptotic bodies (**Figure 1C**). Similarly, TUNEL staining demonstrated that the nuclei of resveratrol-treated U251 cells displayed more frequent and stronger green fluorescence labeling than their control counterparts, whereas these findings were not replicated in LN428 cells (**Figure 1D**).

### Mitochondrial alteration in resveratrol-treated U251 cells

**Figure 2:**
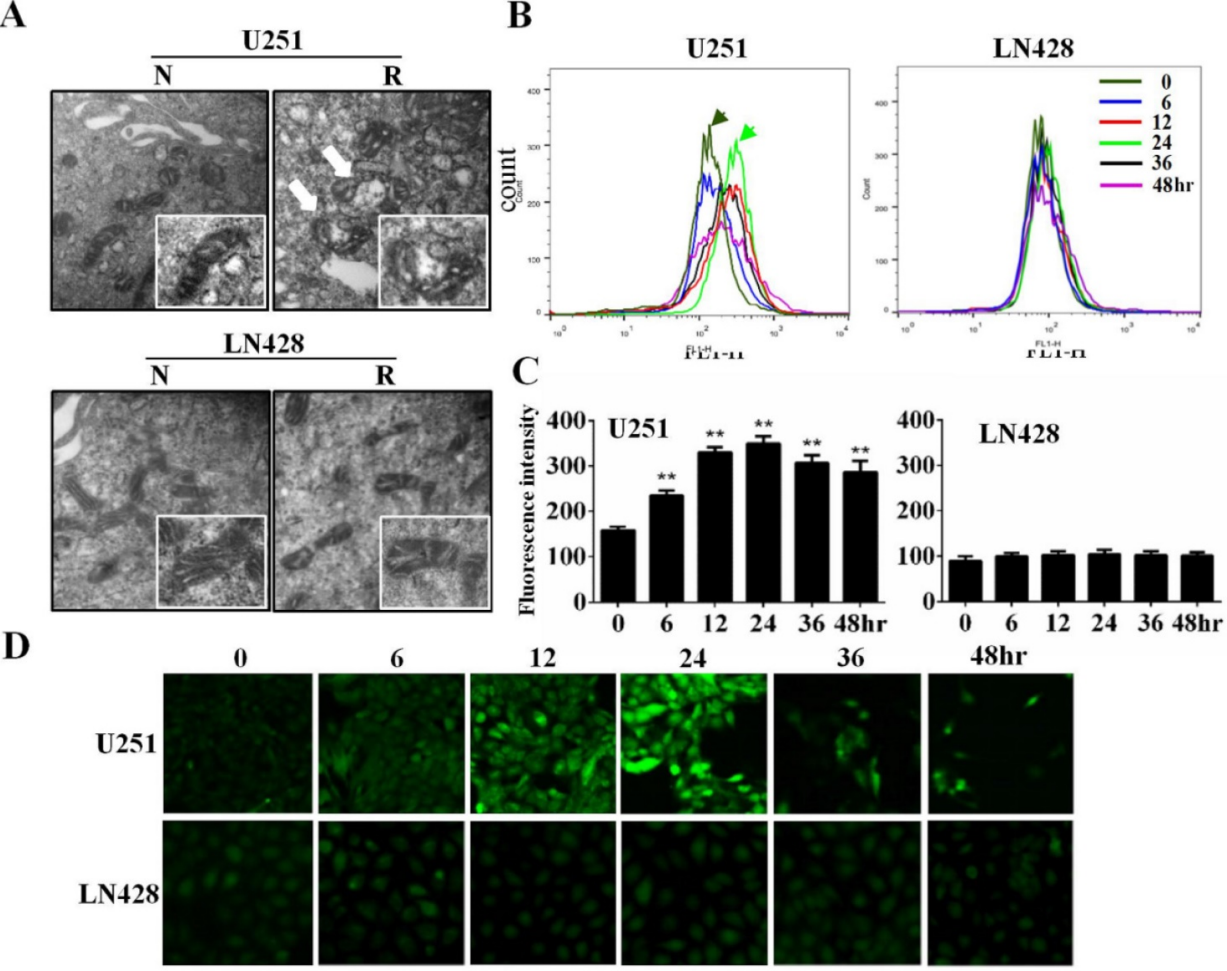
** Mitochondrial spheroid formation and reactive oxygen species (ROS) accumulation in resveratrol-sensitive U251 cells.** (**A**) Transmission electron microscopic examination (×40,000) of the double membrane-defined mitochondrial spheroids (white arrow) in resveratrol-treated U251 cells. N, without resveratrol treatment; R, treated with 100 μM resveratrol for 48 h. (**B**) The cells were treated with 100 µM resveratrol for 0, 6, 12, 24, 36, or 48 h and stained with 2′-7′-dichlorodihydrofluorescein diacetate. Variation of the intracellular fluorescence intensity in U251 and LN428 cells was determined using flow cytometry. (**C**) Fluorescence intensity analysis of U251 and LN428 cells. (**D**) ROS levels in U251 and LN428 cells were measured using fluorescence microscopy. All data are presented as the mean ± SD of three independent experiments. Compared with the 0 h sample, *, *P* < 0.05; **, *P* < 0.01.

Transmission electron microscopy illustrated that in comparison with the intact mitochondria of control cells, double membrane-defined mitochondrial spheroids were commonly observed in resveratrol-treated U251 cells (**white arrow**) but not in LN428 cells treated under the same experimental condition (**Figure 2A**).

### Resveratrol increased ROS levels in U251 cells but not in LN428 cells

Flow cytometry revealed that compared with the basal fluorescence intensity level at 0 h, ROS generation as reflected using relative fluorescence units was significantly increased (*P* < 0.01) in resveratrol-treated U251 cells at 6 (234.04 ± 9.021), 12 (329.40 ± 11.35), 24 (349.09 ± 11.96), 36 (306.17 ± 10.82), and 48 h (285.85 ± 9.701). Conversely, no obvious difference of fluorescence intensity was identified between untreated and resveratrol-treated LN428 cells (*P* > 0.05; **Figure 2B and 2C**). The fluorescence microscopy findings were in accordance with the flow cytometry results in terms of time-dependent increases of DCF fluorescence intensity in resveratrol-treated U251 cells but not in LN428 cells (**Figure 2D**).

### Superoxide dismutase-2 (SOD2) and catalase (CAT) downregulation in U251 cells

**Figure 3:**
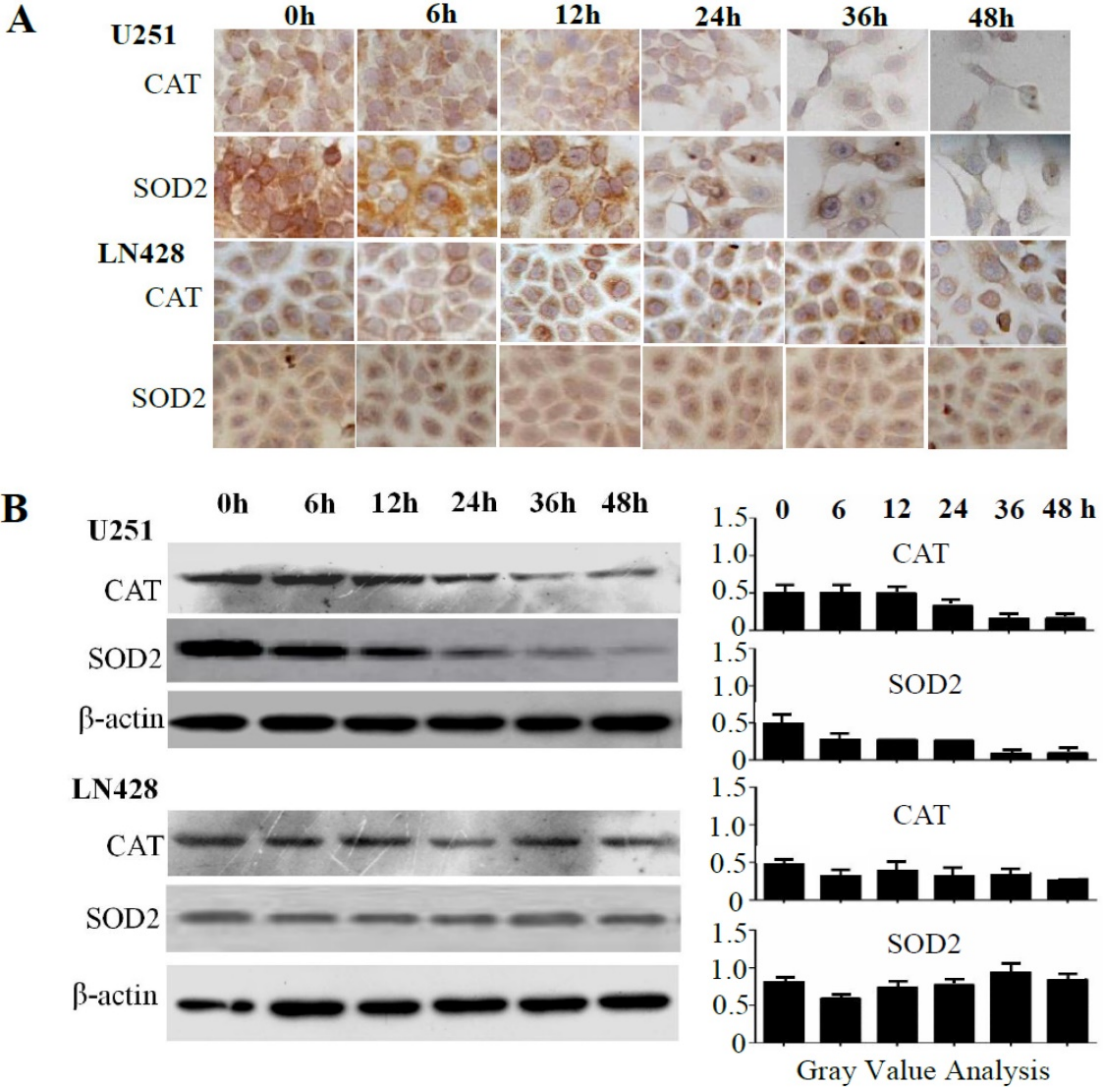
** Sequential analyses of superoxide dismutase-2 (SOD2) and catalase (CAT) levels in resveratrol-treated U251 and LN428 cells.** (**A**) Immunocytochemical evaluation of SOD2 and CAT expression in U251 and LN428 cells treated with 100 µM resveratrol for 0, 6, 12, 24, 36, or 48 h. (**B**) Western blot and gray density analyses of SOD2 and CAT expression in U251 and LN428 cells. β-actin was used as the quantitative control.

The influence of resveratrol on SOD2 and CAT expression was elucidated via immunocytochemical (ICC) staining (**Figure 3A**) and western blotting (**Figure 3B**). The results revealed time-dependent decreases of SOD2 and CAT levels in resveratrol-treated U251 cells, whereas their levels remained relatively stable in the presence of resveratrol in LN428 cells.

### Activated caspase-9 and caspase-3 levels in resveratrol-suppressed U251 cells

**Figure 4:**
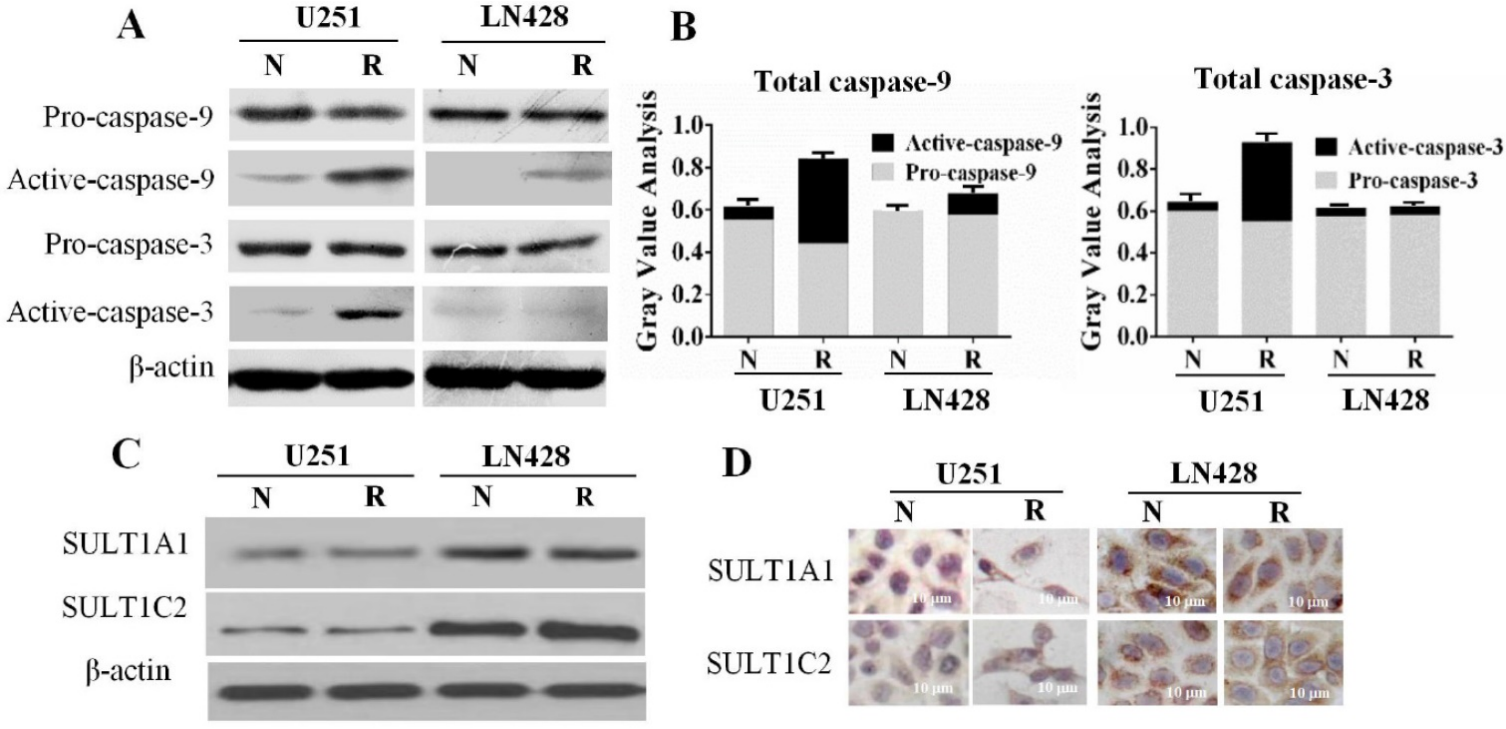
** Caspase-9 and caspase-3 levels and sulfotransferase 1A1 (SULT1A1) and SULT1C2 expression patterns in untreated and resveratrol-treated U251 and LN428 cells.** (**A**) Western blot evaluation of pro-caspase-9, pro-caspase-3, activated caspase-9, and activated caspase-3 U251 and LN428 cells cultured normally (N) and in the presence of 100 µM resveratrol for 48 h (R). (**B**) Fractionation of pro-caspase-9, pro-caspase-3, activated caspase-9, and activated caspase-3 in normally cultured (N) and resveratrol-treated (R) U251 and LN428 cells according to the western blot results. (**C-D**) Western blot and immunocytochemical demonstration of SULT1A1 and SULT1C2 downregulation in U251 cells compared with that in LN428 cells before (N) and after treatment with 100 µM resveratrol for 48 h (R).

The relevance of the mitochondria-mediated apoptotic pathway to resveratrol-induced apoptosis was elucidated by examining the levels of pro-caspase-9 and pro-caspase-3 as well as their enzymatically cleaved forms (activated caspase-9 and caspase-3) in U251 and LN428 cells. Western blotting (**Figure 4A and 4B**) illustrated that overall caspase-9 and caspase-3 levels were decreased by 37.6 and 42.8%, respectively, in 100 μM resveratrol-treated U251 cells. However, whereas pro-caspase-9 and pro-caspase-3 levels were slightly decreased by resveratrol treatment, those of activated caspase-9 and caspase-3 were increased by 6.15- and 7.67-fold, respectively, compared with those in control cells. Meanwhile, pro-caspase-3 and activated caspase-3 levels were largely unchanged in resveratrol-treated LN428 cells. Activated caspase-9 was undetectable in normally cultured LN428 cells, whereas this protein comprised 14.7% of the total caspase-9 pool in resveratrol-treated cells.

### Differential SULT1A1 and SULT1C2 expression in GBM cells

SULTs, the major metabolic enzymes for trans-resveratrol in human cells, have a negative correlation with chemosensitivity 18. Therefore, the expression of the SULT1A1 and SULT1C2 isoenzymes in the two GBM cell lines in the presence and absence of resveratrol was examined. The results of western blotting (**Figure 4C**) demonstrated that SULT1A1 and SULT1C2 were expressed in normally cultured U251 and LN428 cells, and their levels remained almost unchanged after resveratrol treatment. ICC staining revealed that SULT1A1 and SULT1C2 staining was weakly positive (+) in both normally cultured and resveratrol-treated U251 cells. Conversely, SULT1A1 was strongly positive (+++) and SULT1C2 was moderately positive (++) in LN428 cells irrespective of resveratrol treatment (**Figure 4D**).

## Discussion

Intracellular ROS-induced oxidative damage is one of the biological effects of anti-cancer drugs 19-21, as reflected by mitochondrial swelling 22 and spheroid mitochondrion formation 23. Resveratrol has multifaceted biological activities including anti-oxidant and anti-cancer effects 24. Our previous studies revealed that resveratrol has little influence on the growth of normal cells 25, suggesting that the molecular elements related with the effects of resveratrol may be altered in cancer cells, resulting in different biological consequences including oxidative stress. To elucidate this speculation, it is necessary to select resveratrol-sensitive and resveratrol-insensitive cancer cells and measure ROS levels and oxidation-related events after resveratrol treatment.

To date, no efficient therapeutic regimen has been developed for patients with GBM. The nontoxic properties of resveratrol and its ability to cross the blood-brain barrier 26 support the potential usefulness of this polyphenol compound in the management of brain malignancies. However, only certain GBM cell lines, such as U251 cells, are sensitive to resveratrol. Because oxidation-induced mitochondrial spheriods were commonly found in resveratrol-suppressed ovarian cancers 16, we speculated that such biological events may exist in resveratrol-treated GBM cells and that they are related to the sensitivity to this drug. Therefore, the oxidative statuses of resveratrol-sensitive U251 cells and resveratrol-resistant LN428 cells were elucidated by sequentially measuring ROS levels during 48 h of resveratrol treatment. The results demonstrated that ROS levels were elevated in resveratrol-treated U251 cells in a time-dependent manner, whereas ROS levels remained low and stable in resveratrol-treated LN428 cells. These findings revealed the ability of resveratrol to increase oxidative stress in sensitive GBM cells and revealed the positive correlation of ROS levels with resveratrol sensitivity. The distinct spheroid mitochondrion formation found in resveratrol-treated U251 cells but not in drug-treated LN428 cells further supported this notion because this structural alteration is regarded as the consequence of ROS-induced oxidative damage and it could induce mitochondrial dysfunction 27, 28.

The cellular redox balance is typically maintained by a powerful anti-oxidant system in which SOD2 and CAT play active roles 29, 30. Because of the increased ROS levels in resveratrol-sensitive U251 cells but not in resveratrol-resistant LN428 cells, SOD2 and CAT levels in the two cell lines were analyzed before and after resveratrol treatment. SOD2 and CAT levels were similar in the cell lines under normal culture conditions. After drug treatment, SOD2 and CAT expression was downregulated in U251 cells in a time-dependent manner. This phenomenon thus indicates the inefficiency of the anti-oxidant defense system in resveratrol-treated U251 cells, which may explain the resulting ROS accumulation and oxidative damage 31. Anti-oxidation is one of the beneficial effects of resveratrol on normal cells 32, but this effect is less commonly described in cancer cells 15. Our results clearly demonstrated that resveratrol significantly increases ROS levels and downregulates SOD2 and CAT production. Because these events increased oxidative stress and only occurred in resveratrol-sensitive GBM cells, it is reasonable to consider that the redox state of GBM cells is one of the elements explaining their resveratrol sensitivity. It would be worthwhile to investigate other oxidative stress factors such as GPX1 and SOD1 to further support this notion.

ROS accumulation is an early step in mitochondria-mediated apoptosis 30, 32. ROS overproduction causes mitochondrial collapse, leading to the release of cytochrome c and activation of caspase cascades through the conversion of pro-caspase-9 and pro-caspase-3 to their activated forms. Meanwhile, the mitochondria tend to fuse together to form spheroids as a protective response to the increased oxidative stress 16, 31. Therefore, the mitochondrial structure and caspase-3 and caspase-9 expression in the presence and absence of resveratrol were investigated in GBM cells. The results revealed distinct spheroid mitochondrion formation in resveratrol-sensitive U251 cells, and this event appeared within 6 h of resveratrol exposure and before the onset of apoptosis. In accordance, the fractions of activated caspase-9 and caspase-3 were remarkably increased in resveratrol-treated U251 cells. It has been recognized that the release of cytochrome c and activation of the caspase-mediated apoptosis cascade are linked to ROS-induced mitochondrial damage 33. These findings thus provide further evidence of resveratrol-enhanced oxidative stress and its correlation with growth suppression and apoptosis in GBM cells. We also noticed that despite being undetectable in control cells, activated caspase-9 comprised 14.7% of the total caspase-9 pool in resveratrol-treated LN428 cells, a similar level as that (10.3%) found in normally cultured U251 cells. Because the fraction of activated caspase-9 was increased by 6.15-fold accompanied by the appearance of mitochondrial spheroids in resveratrol-treated U251 cells, it is possible that the biological effects of resveratrol on LN428 cells are limited, and induction of activated caspase-9 in these cells may not be sufficient to promote apoptosis.

Resveratrol suppresses cancer cell growth without affecting normal cells because the availability of resveratrol in normal cells is well controlled by efficient biotransformation machinery operated by metabolic enzymes such as SULTs 8, 17, 24. Alternatively, SULT levels are negatively correlated with the anti-cancer efficacy of resveratrol 34, including oxidative stress as described in the current study. For this reason, SULT1A2 and SULT1C2 expression patterns were assessed in control and resveratrol-treated U251 and LN428 cells. The results revealed that basal SULT1A1 and SULT1C2 expression was lower in U251 cells than in LN428 cells, and this situation was unchanged after resveratrol treatment. These results suggest that the decreased metabolic efficiency in U251 cells may increase the intracellular bioavailability of resveratrol and therefore result in oxidative damage instead of anti-oxidant effects. SOD2 and CAT downregulation in resveratrol-treated U251 GBM cells can aggravate oxidative stress, although the underlying mechanism leading to their reduction remains to be investigated.

Taken together, this study demonstrated distinctive growth arrest and apoptosis in resveratrol-treated U251 cells accompanied by remarkably increased ROS levels, caspase-9 and caspase-3 activation, spheroid mitochondrion formation, and reduced SOD2 and CAT production. By contrast, those events did not occur in resveratrol-treated LN428 cells. These findings thus indicate that oxidative stress is one of the cancer-suppressive effects of resveratrol. In this context, ROS levels represent a new parameter for predicting the efficacy of resveratrol against GBMs.
